# A simple coculture system shows mutualism between anaerobic faecalibacteria and epithelial Caco-2 cells

**DOI:** 10.1038/srep17906

**Published:** 2015-12-15

**Authors:** Mehdi Sadaghian Sadabad, Julius Z. H. von Martels, Muhammed Tanweer Khan, Tjasso Blokzijl, Giuseppe Paglia, Gerard Dijkstra, Hermie J. M. Harmsen, Klaas Nico Faber

**Affiliations:** 1 Departments of Medical Microbiology, University of Groningen, University Medical Center Groningen, Groningen, The Netherlands; 2 Departments of Gastroenterology and Hepatology, University of Groningen, University Medical Center Groningen, Groningen, The Netherlands; 3 Departments of Laboratory Medicine, University of Groningen, University Medical Center Groningen, Groningen, The Netherlands; 4 Istituto Zooprofilattico Sperimentale di Puglia e Basilicata, Foggia, Italy; 5 Center for Systems Biology University of Iceland Reykjavik, Iceland

## Abstract

Most gut bacteria are obligate anaerobes and are important for human health. However, little mechanistic insight is available on the health benefits of specific anaerobic gut bacteria. A main obstacle in generating such knowledge is the lack of simple and robust coculturing methods for anaerobic bacteria and oxygen-requiring human cells. Here, we describe the development of a coculture system for intestinal Caco-2 cells and an anaerobic symbiont, *Faecalibacterium prausnitzii*, making use of 50 mL culture tubes. *F. prausnitzii* was grown in 40 mL YCFAG-agar with glass-adhered Caco-2 cells placed on top in 10 mL DMEM medium. Grown for 18–36 h in a humidified incubator at 37 °C and 5% CO_2_, coverslip-attached Caco-2 cells promoted growth and metabolism of *F. prausnitzii*, while *F. prausnitzii* suppressed inflammation and oxidative stress in Caco-2 cells. *F. prausnitzii* did not compromise Caco-2 cell viability. Exogenously added porcine mucin also promoted growth of *F. prausnitzii*, suggesting that it may be part of the mechanism of Caco-2-stimulated growth of *F. prausnitzii*. This ‘Human oxygen-Bacteria anaerobic‘ (HoxBan) coculturing system uniquely establishes host-microbe mutualism of a beneficial anaerobic gut microbe *in vitro* and principally allows the analysis of host-microbe interactions of pure and mixed cultures of bacteria and human cells.

The human gut microbiome is increasingly recognized as an important determinant for human health, affecting a variety of gut, metabolic, neurological and psychological disorders^1^. Gut microbiota provide essential nutrients and anti-inflammatory compounds to the host and confine expansion of pathogens^2^,^3^,^4^. High-throughput sequencing techniques have uncovered the high complexity of the gut microbiome and the composition changes during ageing and disease^5^,^6^. The healthy gut microbiome contains 500–1,000 different bacterial species and their collective genomes (metagenome) encode at least a 100-fold more genes compared with the human genome^7^,^8^,^9^. Only a small fraction of these bacterial species are cultured *in vitro* and even more challenging is to coculture gut bacteria and human cells^10^,^11^. The main obstacle in a host-microbiome coculture system is that most (>90%) gut bacteria are obligate anaerobes that die quickly when exposed to atmospheric conditions (21% O_2_), while human cells depend on oxygen.

*Faecalibacterium prausnitzii* is an obligate anaerobe that may represent up to 25% of all bacteria in the healthy gut^12^. *F. prausnitzii*-excreted products and cell extracts suppress inflammatory signaling in intestinal epithelial (Caco-2) cells *in vitro*, as well as in 2,4,6-trinitrobenzenesulphonic acid (TNBS)-induced colitis in mice *in vivo*^13^. Moreover, human intestinal inflammation is associated with decreased numbers of *F. prausnitzii* and low numbers of this bacterium predispose for post-operative ileal recurrence of Crohn’s disease^13^,^14^,^15^,^16^. Among anaerobes, *F. prausnitzii* has the unique ability to grow close to the intestinal epithelium in the oxic-anoxic interphase of the gut^17^,^18^,^19^. However, it remains elusive whether direct mutualism exists between this gut microbe and intestinal epithelial cells.

In this study, we set out to develop a coculture system for oxygen-requiring human gut epithelial (Caco-2) cells and an anaerobic gut bacterium (*F. prausnitzii*).

## Results and Discussion

**Figure 1:**
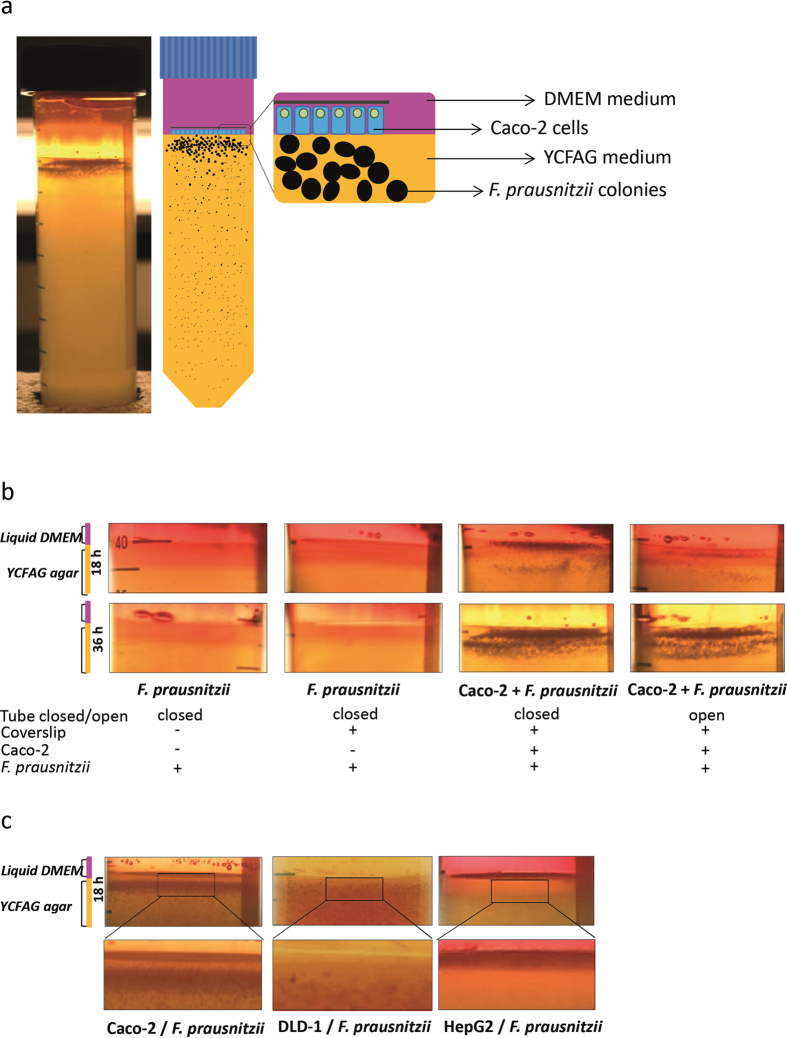
Human intestinal cells promote growth of *F. prausnitzii* in the HoxBan system. (**a**) Schematic drawing of the HoxBan coculture system with *F. prausnitzii* growing in solid YCFAG agar overlaid with liquid DMEM medium and Caco-2 cells on coverslips facing the agar. (**b**) Pictures documenting *F. prausnitzii* colony formation in the absence and presence of Caco-2 cells after 18 h (top panel) and 36 h (bottom panel) in air-open and air-tight culture tubes. (**c**) *F. prausnitzii* colony formation after 18 h coculture with Caco-2 (left), DLD-1 (middle) and HepG2 cells (right).

The ‘Human oxygen-Bacteria anaerobic’ (HoxBan) system developed in this study establishes coculturing of glass-adherent human cells in liquid medium and anaerobic bacteria in solid agar medium for over 24 h and allows the analysis of cell growth, transcriptome and exo-metabolome of cocultured cells. A detailed protocol is given in the supplementary/online section. In short, hand warm (~40 °C) agar medium was inoculated with *F. prausnitzii* starter cultures and aliquots of 40 mL were allowed to solidify in 50 mL Falcon centrifugation tubes. Caco-2 cells grown on coverslips were placed up-side-down on top of the *F. prausnitzii*-containing YCFAG agar and overlaid with DMEM medium. The HoxBan coculture tubes (schematically drawn in Fig. 1a) were placed either with a loose (air-open) or tightly-closed cap (air-closed) in a standard humidified incubator at 37 °C, 5% CO_2_ and atmospheric O_2_. *F. prausnitzii* colony formation, transcriptional adaptations of Caco-2 cells and excreted metabolites in the liquid medium were analyzed after 18–36 h of coculture.

**Figure 2:**
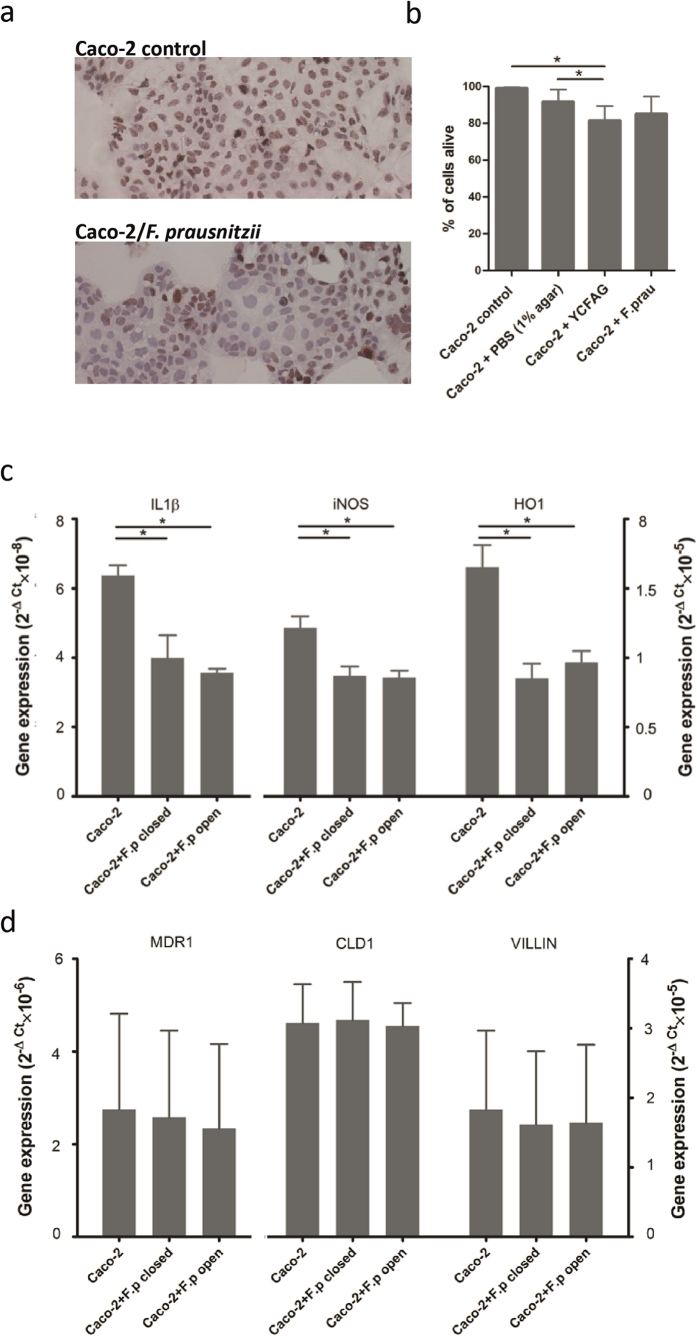
*F. prausnitzii* suppresses expression of inflammatory and oxidative stress markers in Caco-2 cells. (**a**) Ki-67 staining of the Caco-2 cells after 24 h monoculture (top panel) or HoxBan coculture with *F. prausnitzii* (bottom panel). (**b**) Viability of Caco-2 cells after 18–24 h culture in regular 12-wells plates (Caco-2 control), HoxBan setup with PBS-buffered agar, HoxBan with YCFAG agar without (Caco-2+YCFAG) and with *F. prausnitzii* (Caco-2 + F.prau) (**c**,**d**) Comparison of mRNA levels of IL-1b, iNOS and HO-1 (**c**) and MDR1, Claudin-1 (CLD1) and Villin (**d**) in Caco-2 monocultures and Caco-2-*F. prausnitzii* cocultures after 18 h.

Within 18 h, *F. prausnitzii* formed colonies throughout the 40 mL YCFAG-agar. In the absence of Caco-2 cells, no *F. prausnitzii* colonies formed in the top agar layer, most likely due to penetration of intolerable amounts of oxygen (Fig. 1b, left 2 panels). In contrast, clear and bigger *F. prausnitzii* colonies appeared close to the coverslip-attached Caco-2 cells, both in air-closed and air-open tubes (Figs 1b, 2 panels on the right). Notably, *F. prausnitzii* continued to expand over a total coculture time of 36 h (Fig. 1b, 2 bottom panels on the right). In a similar way, human colonic DLD-1 cells promoted growth of *F. prausnitzii* in the top agar layer close to the coverslip, though bacterial growth was less pronounced compared to the cocultures with Caco-2 cells (Fig. 1c). In contrast to both intestine-derived cell lines, HepG2 (human hepatoma) cells did not stimulate *F. prausnitzii* growth (Fig. 1c), indicating that this effect is cell type-specific.

Caco-2 cells were viable and actively dividing even after 24 h of (co)culturing in the HoxBan system (Fig. 2a). Trypan blue staining revealed that over 80% of the Caco-2 cells were viable after overnight culture in the HoxBan system in the absence or presence of *F. prausnitzii* (Fig. 2b). Viability of Caco-2 cells in the HoxBan system without *F. prausnitzii* was significantly lower when compared to either Caco-2 cells grown in 12-wells culture plates or HoxBan system with PBS-buffered agar instead of YCFAG agar. These data imply that both the position of the glass-adherent Caco-2 cells and the contact with bacterial growth medium slightly reduce Caco-2 cell viability, but that this is not affected by the absence or presence of *F. prausnitzii*. Caco-2 cells harvested after overnight culture with and without *F. prausnitzii* revealed that both Il-1β and iNOS mRNA levels were significantly reduced (p < 0.05) in Caco-2-*F. prausnitzii* cocultures compared to Caco-2 mono-cultures (Fig. 2c). A similar effect was observed for the oxidative stress marker heme oxygenase 1 (HO-1; Fig. 2c), In contrast, mRNA levels of villin (a marker for intestinal epithelium), claudin-1 (marker for barrier function) and the multidrug resistance protein-1 (cytoprotective substrate pump) in Caco-2 cells were all similar when cultured in the absence or presence of *F. prausnitzii* (Fig. 2d). These results indicate that expansion of *F. prausnitzii* close to the Caco-2 cells has both anti-inflammatory and anti-oxidant effects, while intestinal barrier functions are maintained.

**Figure 3:**
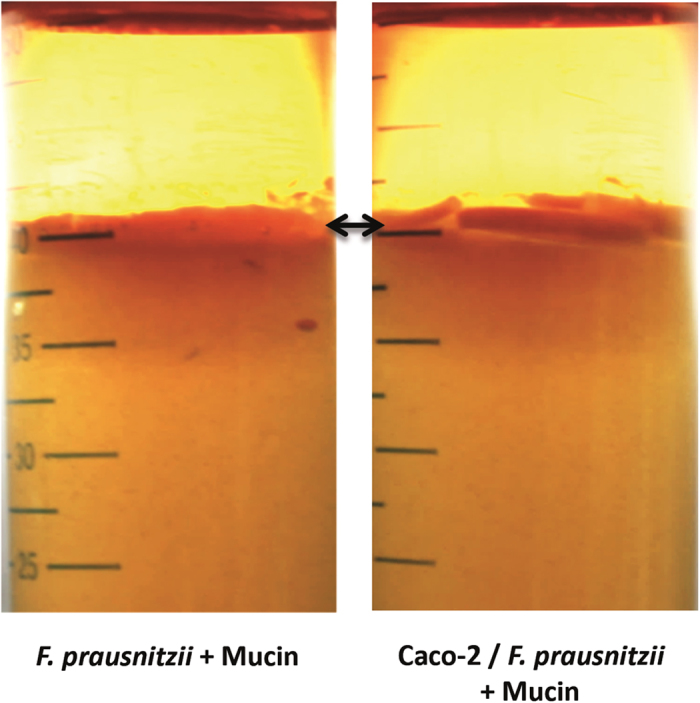
Mucin promotes *F. prausnitzii* growth in the HoxBan system. Pictures documenting *F. prausnitzii* growth when porcine mucin is added on top of the YCFAG agar (indicated by the arrow) in the absence (left) and presence (right) of Caco-2 cells after 18 h culturing.

*F. prausnitzii* has been shown to be able to grow close to the intestinal epithelium adhered to, or even within the mucus layer^9^,^18^,^20^. Caco-2 cells do not express the most dominant intestinal mucin, mucin-2 (encoded by *MUC2*), but have been shown to express *MUC3* and *MUC5A/C*^21^,^22^. To test directly whether mucin may stimulate *F. prausnitzii* growth, porcine mucin type II with 0.8% agar was added on top of the *F. prausnitzii*-containing YFCAG agar in the absence and presence of Caco-2 cells. Mucin alone strongly promoted *F. prausnitzii* growth in the upper layer of the YCFAG agar, which was not affected by the presence of Caco-2 cells (Fig. 3). This indicates that indeed mucin may contribute to the stimulation of *F. prausnitzii* growth by Caco-2 cells, which is a remarkable observation as this gut bacterium is not known to be able to use mucin as carbon and/or energy source^23^.

**Figure 4:**
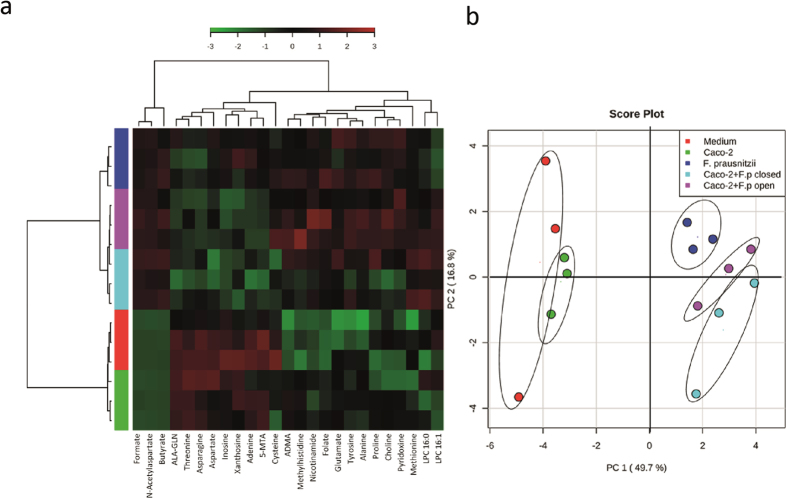
Exo-metabolome analysis of HoxBan mono- and coculture spent medium. (**a**) Hierarchical clustering analysis (HCA) of the top 25 metabolites ranked by the Anova Test. (**b**) Principle component analysis of the metabolites in the medium after 18 h culture. PC1 differentiates between cultures with or without *F. prausnitzii*. PC2 differentiates between *F. prausnitzii* monocultures and the two Caco-2- *F. prausnitzii* cocultures.

**Figure 5:**
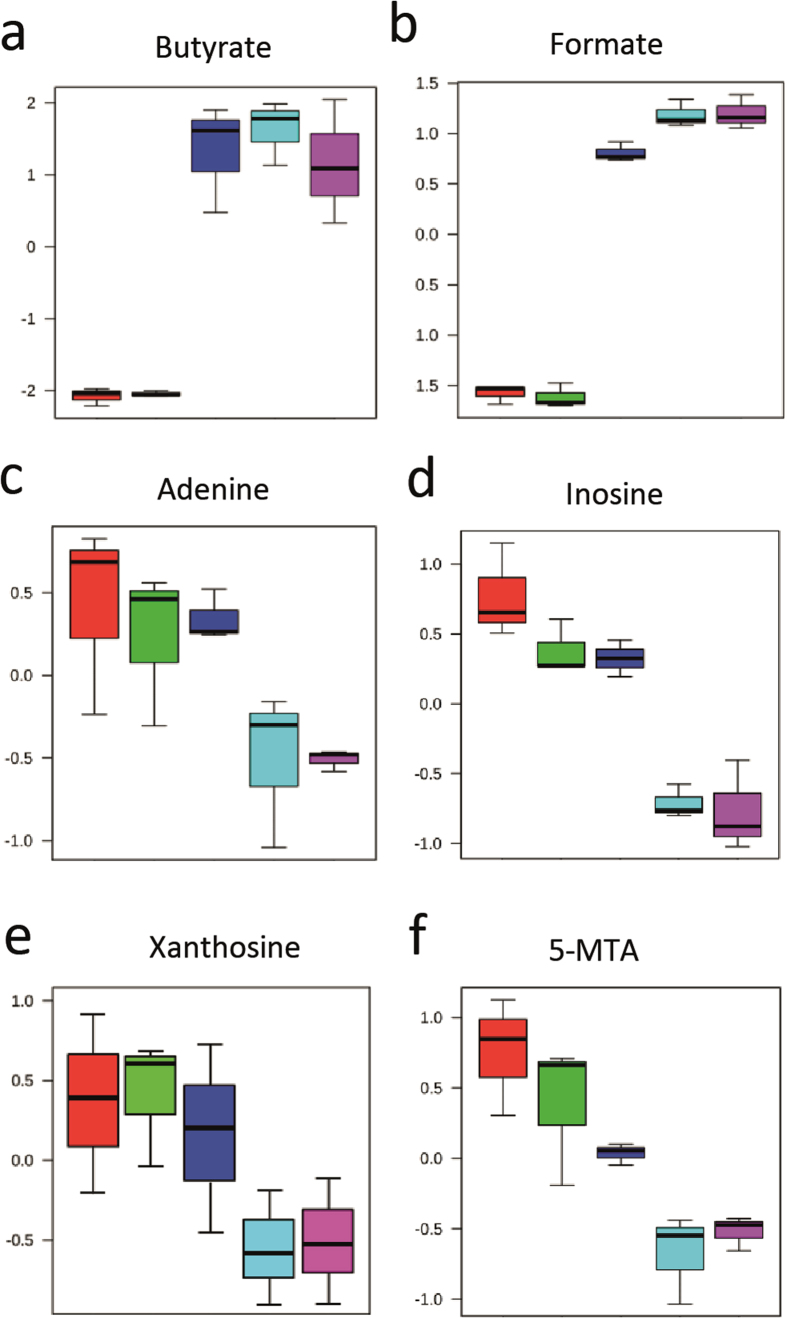
Metabolites that differentiate between HoxBan mono- and coculture spent media. Normalized concentrations of butyrate (**a**), formate (**b**), adenine (**c**), inosine (**d**), xanthosine (**e**) and 5-methylthioadenosine (5-MTA) (**f**) in media after the indicated culture conditions.

To obtain a comprehensive overview of the mutual metabolic effects of *F. prausnitzii* and Caco-2 cells, we performed a metabolome analysis on the liquid medium after 18 hours of (co)culturing Caco-2 cells and/or *F. prausnitzii*, which included short chain fatty acids (SCFAs), hydrocarbons, lipids and amino acids (Fig. 4a). Systematic exo-metabolic changes occurring in different culture condition were assessed using principal component analysis (PCA) (Fig. 4b). The first principal component (PC1) accounts for 49.7% of the total variance and separates the liquid HoxBan culture medium and the Caco-2 monoculture from the *F. prausnitzii* monoculture and the two Caco-2-*F. prausnitzii* cocultures. This indicates that *F. prausnitzii* has the strongest effect on the level of metabolites in the culture medium. Main determinants of PC1 are the SCFAs butyrate and formate that are produced by *F. prausnitzii* (Fig. 5a,b). Other metabolites that associate with PC1 are the essential amino acid methionine and the amino acid-derivative N-acetyl aspartate. Concentrations of all these metabolites are enhanced in HoxBan cultures with *F. prausnitzii*. The second principal component (PC2) accounts for 16.8% of the variance in the data and separates the *F. prausnitzii* monoculture from the two cocultures. Metabolites that contribute to PC2 are formate, adenine and inosine (Fig. 5b–d). Formate levels significantly increased after coculturing *F. prausnitzii* with Caco-2 cells, which is in line with the enhanced bacterial biomass under these conditions. However, butyrate levels did not change upon coculturing *F. prausnitzii* with Caco-2 cells. Butyrate is a preferred energy source of intestinal epithelial cells^24^ and our data suggests that Caco-2 cells consume part of the butyrate produced by *F. prausnitzii*. In turn, this may contribute to the suppression of inflammatory and oxidative stress markers in Caco-2 cells (Fig. 2c).

Together with adenine and inosine, also concentrations of xanthosine and 5-methylthioadenosine were strongly reduced in medium of Caco-2-*F. prausnitzii* cocultures compared to the two monocultures (Fig. 5e,f). These compounds of purine metabolism are required for DNA (cell proliferation) and ATP (energy) synthesis. Their depletion from the medium is likely due to the stimulated growth of *F. prausnitzii*, although a pro-proliferative effect on Caco-2 cells cannot be excluded at this point. On PC2, a slight separation between the open and closed HoxBan Caco-2-*F. prausnitzii* cocultures was observed (Fig. 4b), however, this could not be attributed to significant changes in single metabolites. This indicates that even in the closed condition, 10 mL of DMEM medium carries sufficient oxygen to support the growth of the coverslip-attached Caco-2 cells, which is further supported by the suppressed expression of hypoxia-sensitive HO-1^25^ in both coculture conditions (Fig. 2c). These data show that coculturing Caco-2 cells with *F. prausnitzii* leads to a unique profile of excreted and consumed metabolites that is not simply the cumulative result of the individual cell types, indicating that these cells modify each other’s metabolism.

Thus, the HoxBan coculture system presented here demonstrates for the first time mutualism between oxygen-requiring intestinal epithelial cells (Caco-2) and an obligate anaerobic gut bacterium (*F. prausnitzii*) while both cell types are viable. Several other systems have been developed to study the interaction between human cells and *F. prausnitzii* and attributed anti-inflammatory features to this bacterium^13^,^20^,^26^. However, these systems did not allow the analysis of the effect of human cells on *F. prausnitzii*, which were evidently observed in the HoxBan system. The simplicity of the HoxBan coculture system lies in the use of solid agar medium for growth of the anaerobic gut bacteria, overlaid with liquid medium exposed to air for human cells. The HoxBan is a robust system that can be implemented in almost any molecular biology research laboratory with access to an anaerobic facility and a tissue culture cabinet and incubator. As such, it has great potential to support research to understand the communication between gut microbes and their host. The HoxBan system is readily adaptable to coculture any other anaerobic gut bacterium, as well as complex mixtures of bacteria, with adherent -and potentially also non-adherent- human cell lines or primary cells. Applications of the HoxBan coculture system for other cells than Caco-2 and/or *F. prausnitzii* may require specific optimization in culture conditions, but it holds the universal principle of coculturing oxygen-requiring human cells together with obligate anaerobic bacteria that will foster our understanding of the role of gut bacteria in human health and disease.

## Material and Methods

### Protocol for ‘Human oxygen - Bacteria anaerobic’ (HoxBan) coculture of Caco-2 cells and *F. prausnitzii*

#### Preculture of *F. prausnitzii*

Frozen bacterial stocks were prepared by 1:4 mixing of glycerol (85%) with liquid cultures (optical density at 600 nm (OD_600_) between 1.0–1.5) of anaerobically-grown *F. prausnitzii* strain A2–165 (DSM 17677) at 37 °C in yeast extract, casitone, fatty acids, acetate and glucose (YCFAG) medium^23^,^27^ and stored at −80 °C. Five (5) μl of a *F. prausnitzii* glycerol stock was used to inoculate 5 mL YCFAG broth and incubated for 14–16 hours at 37 °C in an anaerobic incubator until an OD_600_ of approximately 0.8.

YCFAG medium used in this study consists of (all concentration per liter) casitone (10.0 g), glucose (4.52 g), NaHCO_3_ (4 g), CH_3_COONa (2.7 g), K_2_HPO_4_ (0.45 g), KH_2_PO_4_ (0.45 g), NaCl (0.9 g), MgSO_4_·7H_2_O (0.09 g), CaCl_2_·2H_2_O (0.12 g), resazurin (1 mg), hemin (10 mg), biotin (10 μg), cobalamin (10 μg), *p*-aminobenzoic acid (30 μg), folic acid (50 μg) and pyridoxamine (150 μg). Medium was boiled while flushing constantly with CO_2_ and afterwards yeast extract (2.5 g) and cysteine (1 g) were added. Furthermore, short-chain fatty acids (SCFAs) were added: propionate (90 mM), isobutyrate, isovalerate and valerate (10 mM each) (final concentrations). The pH of the YCFAG medium was adjusted to 6.5 − 7 using *MeterLab* pH meter (Radiometer Analytical-France). The medium was autoclaved and filter-sterilized solutions of heat labile thiamine and riboflavin were added afterwards to give the final concentrations of 0.05 μg mL^−1^ each.

#### Preculture of human colon epithelial cell lines

Caco-2 cells were cultured in Dulbecco’s modified Eagle’s minimal essential medium (DMEM) supplemented with 10% fetal calf serum (FCS), penicillin (100 U/mL), streptomycin sulfate (100 μg/mL) and Non-Essential Amino Acids (Gibco® MEM) (1 mL/100 mL). DLD-1 cells were cultured in RPMI 1640 1X + GlutaMAX (Gibco®) and supplemented with 10% fetal calf serum (FCS), penicillin (100 U/mL), streptomycin sulfate (100 μg/mL). Cells were cultured in a humidified incubator at 37 °C in 5% CO_2_ and were seeded at ~50% confluency in 12-well plates containing coverslips and incubated for 48 hours to a confluence of 80–90%. Fresh pre-warmed medium without antibiotics was added 1 hour prior to transferring the cells adherent to coverslips to the HoxBan culture tubes.

### Protocol for the HoxBan coculture

Starting in the anaerobic chamber, one (1) mL of the overnight *F. prausnitzii* preculture was used to inoculate 1000 mL of freshly-autoclaved and cooled-down (~40 °C) YCFAG agar medium containing 1% agar. Aliquots of 40 mL of this inoculum were transferred to sterile 50 mL falcon tubes and allowed to solidify for 30 minutes. The *F. prausnitzii*-inoculated Falcon tube cultures were transferred to a tissue culture cabinet at ambient air and Caco-2 cells on coverslips were placed (up-side-down) on top of the agar and overlaid with 10 mL pre-warmed (37 °C) DMEM medium (without antibiotics), after which the co-cultures were placed in a humidified incubator at 37 °C and 5% CO_2_ for 18–36 hours. The screw caps of the Falcon tubes were either tightly closed (to maintain maximum anaerobic conditions for *F. prausnitzii*) or kept loosely tightened (to allow oxygen exposure for Caco-2 cells). Control conditions were: 1) YCFAG-agar without *F. prausnitzii* inoculum; 2) *F. prausnitzii*-inoculated cultures with coverslips without Caco-2 cells and 3) *F. prausnitzii*-inoculated cultures with human HepG2 cells on coverslips (growth conditions detailed below). Experiments were performed 3 times (N = 3) in triplicate tubes for each condition, with a total of 9 tubes per condition.

#### *F. prausnitzii* growth rim visualization

Visualization of *F. prausnitzii* colony formation in the YCFAG agar close to the coverslips with or without Caco-2 cells was performed using a digital camera (Canon EOS 450D) and the obtained images were processed using Digital Photo Professional software (Canon) without any qualitative and quantitative changes to the raw images.

#### Mucus layer preparation

The mucus layer was prepared essentially as described before by boiling Milli-Q water containing 5% porcine mucin type II (Sigma Aldrich, St. Louis, MO, USA) and 0.8% agar^20^. The solution was autoclaved after adjusting the pH to 6.8. One (1) mL of mucin solution was allowed to solidify on top of the YCFAG-agar, after which coverslips with or without Caco-2 cells and/or DMEM were applied followed by standard HoxBan culture conditions.

#### Harvesting of Caco-2 cells and analysis (Q-PCR and Ki-67 staining)

At the end of the coculture experiment, Caco-2 cells adherent to coverslips were removed from the HoxBan coculture tubes and total RNA was isolated using Trizol according to the suppliers protocol (Sigma-Aldrich). RNA concentrations were determined using a NanoDrop 1000 spectrophotometer (Thermo Fisher Scientific, Wilmington, DE, USA). Reverse transcription polymerase chain reaction (RT PCR) was performed as described^28^. Quantitative PCR (qPCR) for the inducible isoform of nitric oxide synthase (iNOS-NOS2), interleukin-1 beta (IL-1β) and heme oxygenase 1 (HO-1) were performed. Primers (Invitrogen) and probes (Eurogentec) were designed using Primer Express 2.0 software (Applied Biosystems). Details of primers and probes are given in Supplementary Table S1. Q-PCR conditions were as described by Blokzijl *et al*.^28^. Fluorescence was measured using 7900 HT Fast Real-Time PCR system (Applied Biosystems). Each sample was analyzed in duplicate by ABI PRISM Sequence Detector software, version 2.1. Expression of the gene of interest was normalized to 18S^29^. Cell proliferation was assessed by a nuclear Ki-67 staining using a rabbit polyclonal antibody directed against Ki-67 (dilution 1:1000 60 minutes at 25 °C) based on manufacturers protocol (Monosan; Netherlands).

#### Trypan blue staining

Trypan blue solution (0.2%) was added for one minute to the Caco-2 cells adherent to coverslips. After removing the trypan blue solution, the cells were fixed immediately with 4% paraformaldehyde (10 minutes at 4 °C), rinsed 4 times with PBS and mounted on glass slides. Trypan blue-negative (viable) cells were quantified microscopically.

#### Metabolome analysis by Liquid Chromatography-Mass Spectrometry

At the end of the (co)culture experiments the liquid medium on top of the HoxBan cultures was collected (~10 mL) and polar metabolites were analyzed and quantified by ultra-performance liquid chromatography (UPLC Acquity, Waters, Manchester, UK) coupled in line with a quadrupole-time-of-flight hybrid mass spectrometer (Synapt G2, Waters, Manchester, UK) as previously reported^30^. All materials used in the UPLC-MS experiments were purchased from Sigma-Aldrich (Germany) and were of analytical grade or higher purity. For the analysis of targeted metabolites, data were processed using TargetLynx (Waters) while for untargeted analysis MarkerLynx (Waters) was used to integrate and align MS data points and convert them into exact mass retention time pairs. The identity of metabolites was established by comparison of accurate mass measurements and tandem mass spectrometry information against our in-house database and/or online databases^31^,^32^.

#### Short Chain Fatty Acid analysis

For short chain fatty acid (SCFA) analysis, including lactate, formate, butyrate and acetate, a HPLC Ion Chromatography system (Metrohm AG, Herisau, Switzerland) with a conductivity detector was used as described before^19^.

#### Statistics

Principal Component Analysis (PCA) was performed on all detected metabolites and SCFAs by using MetaboAnalyst^33^. Before PCA, data was normalized by the sum, log transformed and then scaled by using pareto scaling.

One-way Anova test was used to detect metabolites that were significantly different between groups. Hierarchical Clustering Analysis (HCA) was then performed by using MetaboAnalyst^33^ on the top 25 metabolites ranked by the Anova Test. The similarity measure was obtained by applying the Spearman’s rank correlation. The clustering algorithm used was the Ward’s linkage. Differences in the dead/alive cell were calculated using Chi-square test. Differences in the gene expressions were assessed by using the Mann–Whitney U test, tests were two-tailed and p-values of 0.05 or lower were considered significant. Tests were performed with PASW Statistics 22 (SPSS, USA).

## Additional Information

**How to cite this article**: Sadaghian Sadabad, M. *et al*. A simple coculture system shows mutualism between anaerobic faecalibacteria and epithelial Caco-2 cells. *Sci. Rep*. **5**, 17906; doi: 10.1038/srep17906 (2015).

## Supplementary Material

Supplementary Information
