# Supplementary material for: A simple coculture system shows mutualism between anaerobic faecalibacteria and epithelial Caco-2 cells
Source: Sci Rep. 2015 Dec 15;5:17906. doi: 10.1038/srep17906 (PMC4678368; doi:10.1038/srep17906)
Supplement: Supplementary Information [file srep17906-s1.doc]

**A simple coculture system shows mutualism between anaerobic faecalibacteria and epithelial Caco-2 cells**

Mehdi Sadaghian Sadabad^1, 2^, Julius Z.H. von Martels^2^, Muhammed Tanweer Khan^1^, Tjasso Blokzijl^3^, Giuseppe Paglia^4, 5^, Gerard Dijkstra^2#^, Hermie J M Harmsen^1#^ and Klaas Nico Faber^2#*^.

Supplementary Table S1: primers and probes used in this study

| **Gene** | **Forward primer sequence 5'-3'** | **Reversed primer sequence 5'-3'** | **Probe sequence 5'-3'** |
| --- | --- | --- | --- |
| **IL-1β** | **aca gat gaa gtg ctc ctt cca** | **gtc gga gat tcg tag ctg gat** | **ctc tgc cct ctg gat ggc gg** |
| **iNOS** | **ggc tca aat ctc ggc aga atc** | **ggc cat cct cac agg aga gtt** | **tcc gac atc cag ccg tgc cac** |
| **HO-1**  **CLDN1**  **MDR-1**  **VILLIN** | **gac tgc gtt cct gct caa cat**  **cca cag cat ggt atg gca ata g**  **ggc aaa gaa ata aag cga ctg aa**  **tga ccc tga gac ccc cat c** | **gct ctg gtc ctt ggt gtc atg**  **cag ccc agc cag tga aga g**  **ggc tgt tgt ctc cat agg caa t**  **tca gca gtg atc tgg ctc ca** | **tca gca gct cct gca act cct caa aga g**  **cag tca atg cca ggt acg aat ttg gtc ag**  **cgt gtc cca gga gcc cat cct gt**  **ttg tgg tga agc agg gac acg agc** |
